# An Evaluation of a Smartphone–Assisted Behavioral Weight Control Intervention for Adolescents: Pilot Study

**DOI:** 10.2196/mhealth.6034

**Published:** 2016-08-23

**Authors:** Chad D Jensen, Kristina M Duncombe, Mark A Lott, Sanita L Hunsaker, Kara M Duraccio, Susan J Woolford

**Affiliations:** ^1^ Brigham Young University Department of Psychology Provo, UT United States; ^2^ Cincinnati Children's Hospital Medical Center Cincinnati, OH United States; ^3^ University of Michigan Health System Ann Arbor, MI United States

**Keywords:** obesity, adolescence, weight control, electronic intervention, self-monitoring

## Abstract

**Background:**

The efficacy of adolescent weight control treatments is modest, and effective treatments are costly and are not widely available. Smartphones may be an effective method for delivering critical components of behavioral weight control treatment including behavioral self-monitoring.

**Objective:**

To examine the efficacy and acceptability of a smartphone assisted adolescent behavioral weight control intervention.

**Methods:**

A total of 16 overweight or obese adolescents (mean age=14.29 years, standard deviation=1.12) received 12 weeks of combined treatment that consisted of weekly in-person group behavioral weight control treatment sessions plus smartphone self-monitoring and daily text messaging. Subsequently they received 12 weeks of electronic-only intervention, totaling 24 weeks of intervention.

**Results:**

On average, participants attained modest but significant reductions in body mass index standard score (zBMI: 0.08 standard deviation units, *t* (13)=2.22, *P*=.04, *d*=0.63) over the in-person plus electronic-only intervention period but did not maintain treatment gains over the electronic-only intervention period. Participants self-monitored on approximately half of combined intervention days but less than 20% of electronic-only intervention days.

**Conclusions:**

Smartphones likely hold promise as a component of adolescent weight control interventions but they may be less effective in helping adolescents maintain treatment gains after intensive interventions.

## Introduction

Overweight and obesity are prevalent health conditions among pediatric populations that increase risk for physical, mental, and emotional problems [1,2]. These conditions are common presenting concerns in pediatric primary, secondary, and tertiary health care systems. Furthermore, treatment of overweight and obesity incurs significant burden including taxing health care systems and increasing health care costs [3]. Numerous treatments for obesity have been developed and evaluated for adults, and treatments for children are also becoming more prevalent [4]. However, treatments targeting adolescents are few, and the efficacy of existing treatments is modest [5]. Furthermore, few studies have demonstrated maintenance of treatment gains after in-person interventions conclude [6]. Enhancing the sustainability of treatment effects is important in demonstrating the value of adolescent weight control interventions. Several studies in the adult literature suggest that mobile health technologies are promising methods for promoting weight-loss maintenance [7]. In addition, many treatments for adolescent weight control are only available in research or hospital settings. Mobile health interventions may extend the reach of behavioral weight-loss interventions [8], an important aim in addressing obesity incidence and morbidity.

One key component of successful weight control is consistent self-monitoring. Although numerous studies have documented the importance of self-monitoring [9], adolescents are generally poorly adherent to this practice [10,11]. Studies have demonstrated that adolescents are more likely to adhere to self-monitoring goals when using an electronic device [12]. A separate but related literature suggests that text messaging is a useful tool for enhancing weight-loss outcomes. In the adult literature, numerous studies have demonstrated the efficacy of text only [13,14] and text plus standard behavioral weight control interventions [15,16]. However, text-messaging interventions for adolescent weight control have received less attention. Text messaging is ubiquitous in the lives of adolescents, with recent estimates suggesting that the daily median number of text messages sent by adolescents exceeds 60 [17]. Text messaging technology holds the capability of delivering intervention content in the treatment of adolescent overweight/obesity. One recent study demonstrated that adolescents perceived routine, automatically generated text messages delivered as part of a weight management program to be acceptable [18].

Smartphone–based behavioral weight control interventions have been evaluated for adults and adolescent studies are beginning to appear. Specifically, Thomas and Wing [19] demonstrated that after 24 weeks of smartphone–based intervention, participants obtained an average weight loss of 10.9 kg with 91% adherence to self-monitoring during the initial 12 weeks of the intervention and 85% adherence in the subsequent 12 treatment weeks. Similarly, Martin et al [20] found that 80% of participants in a smartphone–delivered weight monitoring and feedback intervention lost at least 5% of their body weight after 12 weeks. Furthermore, Burke et al [15] showed that adults who self-monitored via an electronic device demonstrated superior weight loss (63% achieved 5% weight loss) as compared with those who self-monitored via paper and pencil method (46% achieved 5% weight loss). Pretlow et al [21] recently reported that an addiction model intervention delivered by smartphone technology with phone coaching and text messaging produced 7% change in percent over body mass index (BMI) in adolescents.

Existing studies examining smartphone interventions for adolescent weight control are few, and these studies have generally focused on self-monitoring adherence as opposed to weight outcomes [22]. Therefore, the purpose of this study was to determine whether a smartphone–assisted intervention, including self-monitoring support, feedback, and motivational enhancement, could produce changes in weight status and facilitate self-monitoring for overweight/obese adolescents. The smartphone intervention focused specifically on enhancing self-monitoring because this behavior has been shown to be central to adolescent weight control but few adolescents adhere to this practice in weight control interventions [11]. The primary outcome measures in this study were standardized BMI (zBMI), adherence to self-monitoring (percent of days adherent), treatment session attendance, and self-reported satisfaction with the intervention. We also aimed to describe the feasibility of implementing the intervention by integrating narrative feedback from participants to assist others in implementing similar interventions with adolescent populations.

## Methods

### Recruitment

Adolescents were recruited through advertisements posted in schools, pediatricians’ offices, and community health centers. School nurses were also provided study advertisements, which they used to refer potential participants. Interested families responded to the advertisement by phone to be screened by research assistants for eligibility and to schedule an in-person intake session with both the participating adolescent and parent.

### Participants

A total of 16 adolescents aged between 13-17 years with a BMI percentile ≥ 85% and their parent/guardian most responsible for meal preparation were enrolled in the study. Inclusion criteria included: (1) parent/guardian and adolescent consented to participate, (2) the adolescent was aged between 13-18 years, (3) the adolescent exceeded the 85th BMI percentile for age and sex, (4) the adolescent was living at home with their parent/guardian, (5) the adolescent did not have any serious mental illnesses or developmental delays, and (6) participants consented to video recording during the group treatment sessions. Participant demographic characteristics are displayed in Table 1.

**Table 1:** Demographic characteristics of study participants.

		N	%
**Age**			
	13 years	6	38
	14 years	7	44
	15 years	1	6
	16 years	1	6
	17 years	1	6
**Sex**			
	Female	12	75
	Male	4	25
**Educational attainment**			
	Junior high school (7-9 total years)	13	81
	High school (10-12 total years)	3	19
**Race/ethnicity**			
	Non-Hispanic white	9	56
	Hispanic/Latino	4	25
	Other	3	19
**Parent education level**			
	High school graduate	1	6
	Attended college	2	12
	Associate’s degree	3	18
	College graduate	7	46
	Graduate degree	3	18
**Parent monthly income**			
	Range	$2000-$16,666	
	Average	$6151.45	

### Procedure

Study procedures were approved by the Brigham Young University Institutional Review Board for Human Subjects. Participants attended an initial in-person meeting where they provided informed consent/assent and completed an initial assessment of height and weight conducted by trained research assistants and baseline questionnaires. Study assessments occurred on 4 occasions over a 1-year period including: (1) at intake (time 1; N=16), (2) after completion of the 12-week group intervention (time 2; N=14), (3) after the 12-week electronic-only intervention (time 3; N=14), and (4) 1 year after the first treatment session (time 4, N=10) with final study assessments concluding in June 2014. Missing data resulted exclusively from participant’s failure to respond to invitations to complete study assessments. At the initial treatment session, each adolescent participant was provided an electronic device for self-monitoring (iPhone 4) with a diet/physical activity app installed (Daily Burn Tracker) for the duration of the intervention period (24 weeks). Unlimited data/voice/text plans were provided to participants free of charge. One participant elected to use their own iPhone to avoid challenges with duplicate devices. All other participants used the phone supplied by the researchers exclusively during the 24-week intervention period. After completing the 12-week combined intervention (combined treatment), electronic self-monitoring and text messaging continued for an additional 12 weeks. Self-monitoring was selected as the primary goal of the electronic-only intervention period because of its documented importance in facilitating adolescent weight control.

### In-Person Intervention Description

Participants attended a 12-week group weight control program led by clinical psychology doctoral students under the supervision of licensed psychologist, with parents and adolescents attending separate group meetings in adjoining rooms. Pediatric psychologists delivered the intervention because of their expertise in weight-related health behavior change. Group meeting duration was 75 minutes. Weekly treatment supervision meetings were held to ensure adherence to the treatment protocol. Group sessions were standardized by using a 12-week modular behavioral weight control program developed by Jelalian et al [23] that included important components of weight management including self-monitoring, portion control, problem solving, stimulus control, emotional eating, and physical activity. Each parent/adolescent dyad also received 15 minutes of individual family intervention every 4 weeks after group sessions. During individual family meetings, motivational interviewing was used to assess motivation and to problem solve how to overcome barriers to treatment.

### Electronic Self-monitoring and Text Messaging Intervention Description

The smartphone–based treatment component consisted of 2 parts: electronic self-monitoring and human-generated text messaging. Consistent with previous studies conducted with adults, participants were instructed to record all meals/snacks and physical activity electronically. DailyBurn Tracker, a commercially available smartphone app, was used for self-monitoring. This method simplified self-monitoring by allowing participants to search for foods in a database that contained nutrition information or to scan barcodes on food labels to locate nutrition information. Moreover, participants could store unlimited “favorite” foods in DailyBurn. An intuitive user interface in DailyBurn allowed participants to organize foods consumed by meal, indicating quantity and summing dietary characteristics (e.g., total calories, total fat, and so forth). Physical activity was tracked using the same app. Participants received real-time feedback regarding their goal attainment using DailyBurn. Specifically, participants could view a visual analog scale demonstrating percentage of their caloric goals consumed at any time during the day. DailyBurn accounts were created for each participant by study staff, allowing researchers to view participant’s self-monitoring and diet behavior in real time.

Brief text messages were sent to each participant once per day in the evening by graduate and undergraduate research assistants. Text messages served 2 purposes. First, the messages provided feedback regarding participant self-monitoring behavior and progress toward treatment goals (eg, reducing sugar-sweetened beverages). Feedback was based on real-time self-monitoring data viewed in DailyBurn by study staff. Feedback was supportive and encouraged adherence to self-monitoring (eg, “remember to track your breakfast this morning”). Second, text messages contained content designed to reinforce principles addressed in the in-person treatment, including seeking social support, expanding diets to include healthier foods, and altering food environments. These text messages were selected from a library developed by Woolford et al [24] and the messages frequently used motivational interviewing strategies to evoke introspection and encourage autonomous motivation (eg, “What motivates you to eat breakfast every day?” “How can you add fruits or vegetables to your breakfast?”). Participants were not expected to text study staff in return.

### Measures

#### Anthropometric Data

Weight was measured using a digital scale (Seca 869, measured to the tenth of a pound) and height was measured using a portable stadiometer (Seca 217, measured to the eighth of the inch) with participants wearing light clothing and no shoes. The zBMI scores were calculated using the standard CDC formula [25].

#### Adherence to Self-Monitoring and Treatment Attendance

Adolescent’s self-monitoring data were downloaded from DailyBurn to calculate self-monitoring adherence. Consistent with Thomas and Wing [19], days on which participants tracked 2 or more meals were considered self-monitoring adherent days. Treatment attendance was calculated as a percentage of in-person treatment sessions attended.

#### Participant Satisfaction

Satisfaction with the treatment program was measured using the Client Satisfaction Questionnaire [26]. This eight-item scale has demonstrated adequate reliability (α=.83-.93) and validity in previous studies.

#### Semistructured Treatment Exit Interview

At the conclusion of the study, each participant was interviewed about his or her participation experience. Interview questions were open-ended and covered topics including study components that were helpful, changes that could be made to the intervention, and their impressions of using a smartphone as part of the study. For example, interview questions included, “What aspects of the program were helpful to you in managing your weight?” and “What is your opinion about using the smartphone as part of the program?” All interviews were transcribed by a research assistant who did not conduct the interviews.

#### Statistical Analysis Plan

Data analyses were conducted using Stata 13 [27]. Descriptive statistics were calculated for baseline demographic variables and primary study variables (eg, zBMI, self-monitoring, treatment satisfaction, treatment session attendance) including means and standard deviations. Paired samples *t* tests were used to examine change in zBMI over time and estimates of effect size (Cohen’s *d*) were computed. Correlation analyses were used to test associations between self-monitoring adherence/treatment session attendance and change in zBMI. Participants with insufficient data to calculate zBMI at any given time point were excluded from analyses.

## Results

Mean statistics for weight status at each measurement occasion are presented in Table 2. On average, participants were in the obese category at baseline with a BMI% of 95.78 (SD=3.51). A paired samples *t* test comparing zBMI at time 1 to time 2 indicated a significant decrease; *t* (13)=2.22, *P*=.04, *d*=.63. On average, participants attained a 0.08 standard deviation decrease in zBMI. Neither time 3 nor time 4 zBMI was significantly different from baseline. These results are consistent with modest mean weight-loss over the in-person intervention period but subsequent return to baseline levels during the 3-month electronic intervention-only period.

**Table 2:** Changes in participant weight status.

	Time 1, mean (SE^a^), N=16	Time 2, mean (SE), N=14	Time 3, mean (SE), N=14	Time 4, mean (SE) N=10
Body weight (lbs)	175.10 (10.29)	172.53 (11.14)	177.93 (10.27)	175.40 (5.38)
zBMI^b^	1.85 (0.11)	1.74 (0.13)	1.78 (0.13)	1.78 (0.12)

^a^SE: standard error.

^b^zBMI: standardized body mass index.

On average, participants attended 7.5 (62.5%) out of 12 in-person treatment sessions (SD=0.85). A correlation analysis showed no significant association between treatment session attendance and change in weight status between time 1 and time 2 for zBMI. On average, participants monitored at least 2 meals on 48.3% of days during the in-person intervention (12 weeks), with 7 participants monitoring their diet over 50% of in-person intervention days. Participants monitored at least 2 meals on 16.6% of the available days during the electronic-only intervention period. On average, participants tracked at least 30 minutes of physical activity on 14.6% of available days during the 12-week in-person intervention and 4.6% of available days during the electronic-only intervention. Paired samples *t* tests comparing diet and physical activity self-monitoring during in-person and electronic-only intervention periods indicated significant differences (*t* (15)=5.68, *P*<.001, *d=.* 46; *t* (15)=3.67, *P*=.002, *d=*.38, respectively). Correlation analyses examining the association between diet/physical activity self-monitoring and zBMI were not significant.

At completion of the 6-month intervention period Client Satisfaction Questionnaire rating was 20.33 (maximum=22). Participants also completed an exit interview soliciting feedback regarding perceptions of the treatment (see Table 3). Of the 15 adolescents who completed the interview, most described the intervention favorably (N=13; 86.7%), reporting that the intervention “worked well” or was “very helpful.” Specifically, our participants endorsed enjoying learning about nutrition and exercise (N=5; 33.3%) and being able to meet with an expert to have their questions answered (N=3; 20%). Most of our participants reported that they favored meeting in a group over the electronic-only intervention (N=11; 73.3%). Two participants (13.3%) described the intervention unfavorably, stating that they “didn’t get as much help as [they] would have liked.” Overall, half of our participants viewed the intervention’s emphasis on self-monitoring as helpful (N=8; 53%). Despite this positive view of self-monitoring, about half of our participants found the DailyBurn app to be “tedious” and “difficult to use” (N=8; 53.3%).

Most of our participants viewed the text messages that they received from study staff very positively (N=11; 73.3%). Four of our participants (26.7%) stated they wished that they had received more texts. Several participants noted that the text messages were “enthusiastic” and “uplifting” and that they “reminded (them) that (they) were doing a good job.” However, a subset of our participants found the texts to be unhelpful (N=4; 26.7%). When asked what we could do improve our text messages, the most consistent feedback that we received was to include healthy recipes in our text messages (N=8; 53.3%).

**Table 3:** Themes from qualitative treatment exit interviews with illustrative quotations.

Intervention themes	Impressions (N)	Quotations
**Broad impressions**	Positive (13)	*I liked the (intervention); it was helpful to know that there are other kids like me.*
	Negative (2)	*I don’t know how an intervention can be an enjoyable thing! It made me feel like I was doing everything wrong and nobody else has this problem.*
**Group**	Positive (11)	*Sharing what we did with the group was really helpful. I wish we (could have) had more group meetings together.*
	Neutral or negative (4)	*It would have been nice to have two groups...split the older group from the younger group.*
**iPhone**	Positive (9)	*It was easier (to track) because you always have (your phone) with you.*
	Negative (6)	*At times the phone was distracting. I used the phone for other things besides recording calories and exercise.*
**Self-monitoring**	Positive (8)	*Tracking helped me become come more aware, (which) was helpful. (It helped me) focus on healthy choices and making good long-term life choices.*
	Neutral or negative (7)	*The tracking was kind of annoying and I don’t think that I really got help as much as I’d like.*
**DailyBurn**	Positive or neutral (7)	*The only thing that helped was DailyBurn.*
	Negative (8)	*It was a challenge to do DailyBurn because it took time to type (in foods) and find (foods).*
**Text messages**	Positive (11)	*(The texts) made me feel really nice. It reminded me that I was doing a good job.*
	Neutral or negative (4)	*I got annoyed (by the texts) really fast. I’m sorry! But when I’m told to do something I was going to do anyway, I will do the exact opposite of what is asked.*

## Discussion

### Principal Findings

This pilot study was one of the first to use smartphone technology to enhance self-monitoring and deliver tailored behavioral weight control text messages to adolescents as part of a clinical intervention. Similar to previous studies, participants demonstrated modest but significant reductions in zBMI from baseline to the end of the in-person intervention portion of the study, although reductions in zBMI were not sustained over the electronic-only intervention period. These findings suggest that text messaging and electronic self-monitoring may be helpful when paired with behavioral intervention, but that text messaging and electronic self-monitoring may not be effective as standalone treatment. Similarly, Nguyen et al [6] recently found that although adolescents were able to achieve weight loss during the in-person portion of an intervention combining face-to-face treatment plus telephone coaching and text messaging/email contact, they failed to maintain weight loss once the in-person intervention ceased. Although both standalone and adjunctive smartphone interventions (ie, smartphone plus in-person treatment) have been shown to be effective in the adult literature, our results support an adjunctive treatment model.

One potential explanation for our finding that zBMI change did not persist in the electronic intervention-only period is that adolescents were less able to draw on the social support that they received while engaging in the in-person intervention. Most of our participants favored meeting in the group setting, and several of our participants desired increased social connectedness throughout the course of the intervention. Furthermore, studies have shown that social support is an important motivator for individuals who want to lose weight and having social support helps individuals maintain weight loss [28]. Although adult studies have found electronic device interventions to be effective without an emphasis on social support [19], we theorize that social support would be more salient and effective for adolescents. Furthermore, research has shown that electronic health interventions that have a focus on increasing social support have an additive benefit when trying to achieve weight loss [29]. mHealth interventions for weight loss could increase social support for behavior change through social media such as Facebook groups, a method that has been used successfully in young adults [30].

On average, our participants recorded 2 or more meals roughly 1 out of every 3 days. Interestingly, self-monitoring adherence did not predict zBMI change in this study. The overall adherence to self-monitoring observed in our sample is lower than what has been found in previous studies [15]. We found that adherence to diet and physical activity self-monitoring was significantly higher during in-person (vs electronic-only) intervention. These findings suggest that, even when provided with a smartphone and self-monitoring app, adolescents demonstrate relatively low adherence to self-monitoring, particularly when compared with adult studies [19]. One potential explanation for the low self-monitoring adherence observed in our study is that some participants perceived the Daily Burn app as “tedious” and “difficulty to use” which may have deterred some individuals from self-monitoring. Developing less burdensome self-monitoring apps such as those integrating gamification may increase adolescent’s adherence to self-monitoring.

Most of our participants rated their satisfaction with the intervention highly. Adolescents reported perceiving the text messaging and self-monitoring to be helpful components of the intervention. Most participants found the iPhone to be useful for self-monitoring because it was available to them throughout the day. Our participants provided several suggestions for improvement to our weight control program. For example, most of the participants viewed the DailyBurn app as burdensome. Intervention designers might consider incorporating more user-friendly apps to record diet and exercise. Several previous studies have created study-specific smartphone apps to ensure that the app was user-friendly and age-appropriate [31,32]. Consistent with previous studies [24], participants in our study perceived text messages positively, particularly messages that encouraged behavior change (eg, providing novel meal suggestions). They expressed a preference for motivational/supportive messages whereas behavior feedback messages created for this study (eg, “remember to track your diet today”) were less preferred. This finding is consistent with previous qualitative research that has indicated that adolescents may experience ambivalence about receiving directive weight control text messages [33].

Regarding feasibility, our study model requires both in-person interventions (similar to other treatments) in addition to electronic treatment content delivery. Dissemination of our intervention approach is also limited because human-delivered text messages are cost intensive. One way to alleviate this burden is to incorporate automated delivery of text messages [18,34]. Limitations to this study include the small, homogeneous study sample and the lack of a comparison condition. We provided an iPhone with data/voice/text plan to study participants, which limits disseminability of the intervention. However, smartphones are increasingly available to adolescents and many individuals can use their own phones to receive interventions.

### Conclusion

Although participants were able to lose weight during the in-person treatment, they were unable to maintain weight loss during the electronic-only intervention period. One potential explanation for this finding is that our study did not incorporate problem solving, stimulus control, and emotional eating components into the electronic-only intervention, which may have contributed to poor maintenance of weight loss. Future research integrating these components that have been shown to be important in in-person treatments into mHealth interventions is important. Despite these limitations, this study is important because it was one of the first to examine weight status outcomes of a smartphone–assisted behavioral weight control intervention for adolescents. Furthermore, our study provides important formative feedback for development of future smart phone interventions for adolescent weight control.

## Abbreviations

BMI: body mass index
SD: standard deviation
